# Trehalose Protects the Probiotic Yeast *Saccharomyces boulardii* against Oxidative Stress-Induced Cell Death

**DOI:** 10.4014/jmb.1906.06041

**Published:** 2019-09-24

**Authors:** Ji Eun Moon, Wan Heo, Sang Hoon Lee, Suk Hee Lee, Hong Gu Lee, Jin Hyup Lee, Young Jun Kim

**Affiliations:** 1 Department of Food and Biotechnology, Korea University, Sejong 3009, Republic of Korea; 2 Institutes of Natural Sciences, Korea University, Sejong 30019, Republic of Korea; 3 Department of Molecular Medicine, Cell and Matrix Research Institute, Kyungpook National University School of Medicine, Taegu 41566, Republic of Korea; 4 Department of Animal Science and Technology, College of Animal Bioscience and Technology, Konkuk University, Seoul 05029, Republic of Korea

**Keywords:** Trehalose, probiotic, reactive oxygen species, programmed cell death

## Abstract

*Saccharomyces boulardii* is the only probiotic yeast with US Food and Drug Administration approval. It is routinely used to prevent or treat acute diarrhea and other gastrointestinal disorders, including the antibiotic-associated diarrhea caused by *Clostridium difficile* infections. The formation of reactive oxygen species (ROS), specifically H_2_O_2_ during normal aerobic metabolism, contributes to programmed cell death and represents a risk to the viability of the probiotic microbe. Moreover, a loss of viability reduces the efficacy of the probiotic treatment. Therefore, inhibiting the accumulation of ROS in the oxidant environment could improve the viability of the probiotic yeast and lead to more efficacious treatment. Here, we provide evidence that supplementation with a non-reducing disaccharide, namely trehalose, enhanced the viability of *S. boulardii* exposed to an oxidative environment by preventing metacaspase YCA1-mediated programmed cell death through inhibition of intracellular ROS production. Our results suggest that supplementation with *S. boulardii* together with trehalose could increase the viability of the organism, and thus improve its effectiveness as a probiotic and as a treatment for acute diarrhea and other gastrointestinal disorders.

## Introduction

Probiotics are generally defined as live microorganisms with low or no pathogenicity that exert beneficial effects on the health of the host when consumed in adequate amounts [1]. The majority of microorganisms recognized as probiotics are bacteria. However, a non-pathogenic yeast, *Saccharomyces boulardii*, is the only probiotic yeast that has been approved by the US Food and Drug Administration (FDA) for human consumption [2]. Prescription of *S. boulardii* has been clinically approved to treat antibiotic-associated diarrhea (AAD) related to bacterial infections, such as *Clostridium difficile*, which accounts for a third of clinical presentations of AAD [3-7]. The benefits of *S. boulardii* as a probiotic have been confirmed by several clinical studies that have demonstrated its effectiveness in the prevention of acute diarrhea and gastrointestinal disorders in both pediatric and adult populations [8-12].

In aerobic organisms, including *S. boulardii*, reactive oxygen species (ROS) are inevitably generated by internal metabolic processes associated with oxygen respiration. Hydrogen peroxide (H_2_O_2_) is the most abundant ROS in vivo, since it is continuously produced intracellularly as a byproduct of normal aerobic metabolism [13-15]. The accumulation of ROS inflicts oxidative damage upon essential cellular components, such as nucleic acids, lipids, and proteins [13, 16]. In particular, the oxidative damage to lipids and proteins mediated by H_2_O_2_ is considered to be the main causative mechanism of cellular damage contributing to programmed cell death (PCD), which represents an immediate risk to cell viability [17, 18].

Oxidative stress caused by the accumulation of ROS can result in the retarded growth, inhibition of metabolic activity, and ultimately loss of viability of a probiotic microorganism, directly impairing the efficacy of treatment [14, 19, 20]. Recently, a protective effect of polyphenols against the toxic effects of ROS in yeast was reported [21-23]. Thus, it can be hypothesized that under the circumstances of oxidative stress, probiotic yeast might be protected using natural or synthetic antioxidants that would scavenge the ROS, and lead to increased cell viability. In the present study, the non-reducing disaccharide, trehalose (α-D-glucopyranosyl-1,1-α-D-glucopyranoside), was selected as an antioxidant, since it has been reported to possess a strong ROS-scavenging property [17, 24]. Moreover, it was recently reported that trehalose prevents H_2_O_2_-triggered death in human dopaminergic SH-SY5Y cells via mitigation of ROS-dependent endoplasmic reticulum stress [25], which strongly potentiates our hypothesis that trehalose protects the probiotic yeast *S. boulardii* against oxidative stress-induced cell death.

An approach for reducing the oxidative stress faced by probiotic aerobic microorganisms may lead to more efficacious probiotic treatments. Here, we provide evidence that when *S. boulardii* was cultured in the presence of H_2_O_2_, as a model of oxidative stress-induced cellular damage, the organism became less viable. The reduced viability was due to the accumulation of ROS and the activation of caspase-like enzymatic activity, which are hallmarks of PCD [26]. Moreover, we show that trehalose, a commercially available dietary supplement, enhanced the cell viability of *S. boulardii* exposed to oxidative stress, most likely by preventing ROS-mediated PCD. Given that many of the proven health benefits of *S. boulardii* depend on its viability, our data suggest that supplementation with the combination of *S. boulardii* and trehalose may be a more effective treatment for acute diarrhea and other gastrointestinal disorders than supplementation with the probiotic agent alone.

## Materials and Methods

### Yeast Strain and Growth Conditions

All experiments were performed with *S. boulardii* (CNCM I-1079) obtained from LALLEMAND Inc. (Canada). The yeast was grown at 30°C in a standard liquid yeast extract peptone dextrose (YPD) broth (BD Difco, DF0427-17-6) on a rotary shaker at 200 rpm or on YPD agar containing 2% agar.

### Identification of *S. boulardii* by Polymerase Chain Reaction (PCR)

Total genomic DNA was extracted from the yeast cell pellet using a DNeasy Blood and Tissue Kit (Qiagen), according to the manufacturer’s instructions, and was quantified by ultraviolet-visible spectrophotometry. The identification of *S. boulardii* was performed by the PCR-based amplification of the internal transcribed spacer (ITS) region between the rDNA subunits, including the gene that encodes 5.8S rRNA, using primers for ITS2 (forward primer: 5’-AGGTTTTACCAACTGCGGCT-3’; reverse primer: 5’-TCGCCTAGACGCTCTCTTCT-3’) and microsatellite loci using primers for YLR177w (forward primer: 5’-CTTAAA CAACAGCTCCCAAA-3’; reverse primer: 5’-ATGAATCAGCGC ATCAGAAAT-3’). The PCR mixture consisted of 1 μl of genomic DNA, 10 μM dNTPs, 0.5 μM of each primer, 10 × Taq buffer, and Taq polymerase in a final volume of 20 μl. Annealing with primers was performed at 57°C for 45 sec and extension was performed at 72°C for 1 min. After 35 cycles, the PCR products were separated on 2% agarose gels and bands were visualized by ethidium bromide staining.

### Cell Viability Assay

To determine cell viability, the yeast cells from the exponential phase culture were centrifuged, washed with sterile water, and suspended at a final density of 10^8^ cells/ml in 100 mM phosphate buffer (pH 7.0) containing 0.1% glucose and 1 mM ethylenediaminetetraacetic acid with 1 M H_2_O_2_ in the absence or presence of 1 M trehalose (CAS number: 6138-23-4) purchased from Sigma-Aldrich (USA). After incubation for 1 h, the cells were pelleted by centrifugation, washed twice with sterile water, and then suspended in water or a buffer solution, as appropriate. Cells were suspended in sterile water and diluted to a final concentration of 103 cells/ml. A sample (100 μl) of the suspension was spread on YPD agar and incubated at 30°C. Colony-forming units were counted after 24 h.

### Assessment of Metabolic Activity of the Cells

After incubation, the cells were suspended in 10 mM Na-4-(2-hydroxyethyl)-1-piperazineethanesulfonic acid (pH 7.2) containing 2% glucose. The metabolic activity of the cells was estimated with 1 μM FUN-1 stain (100 μM stock in dimethylsulfoxide). Metabolically active cells were observed to contain cylindrical red fluorescent structures in their vacuoles. Cells with little or no metabolic activity had diffuse green cytoplasmic fluorescence and lacked fluorescent intravacuolar bodies; and dead cells exhibited extremely bright, diffuse, green-yellow fluorescence [27]. The metabolic activity of the cells was expressed as a change in the ratio of red (λ = 575 nm) to green (λ = 535 nm) fluorescence. The fluorescence of the cell suspensions was measured 30 min after the addition of FUN-1, using a Zeiss Axiovert 200 inverted microscope (Germany).

### Apoptosis Measurement Using the Terminal Deoxynucleotidyl Transferase-Mediated dUTP-Biotin Nick-End Labeling (TUNEL) Assay

Yeast cells grown to the early exponential phase at 30°C were exposed to 2 mM H_2_O_2_ for 1 h in the absence or presence of 100 mM trehalose, then harvested for apoptosis measurement. A TUNEL assay was performed to determine the occurrence of apoptosis [28]. *S. boulardii* cells were washed twice with phosphate-buffered saline (PBS) and fixed with a solution of 4%paraformaldehyde in PBS for 30 min at 20°C. Lyticase was added in the cells which were incubated for 1 h at 37°C. The cells were rinsed with PBS and then incubated with 0.1% Triton X-100 for 2 min on ice. The cells were rinsed in PBS and labeled, using a solution of the label (fluorescein) and enzyme solutions from an *In Situ* Cell Death Detection Kit (Roche Applied Sciences, Germany). Appropriate controls were labeled only with the label solution. The cells were next incubated for 1 h at 37°C in a humidified atmosphere in the dark, then rinsed in PBS. Staining of the cells was visualized by fluorescence microscopy at excitation and emission wavelengths of 488 and 520 nm, respectively.

### Assessment of YCA1 Caspase Activity

Activated YCA1 caspase activity was detected in *S. boulardii* cells, after treatment with H_2_O_2_ and trehalose as described in section 2.5, using a CaspACE FITC-VAD-FMK *in situ* Marker (Promega), according to the manufacturer’s specifications. The yeast cells were exposed to the reagents of the apoptosis detection kit at 30°C for 20 min before observing and counting them under a fluorescence microscope with excitation and emission wavelengths of 488 and 530 nm, respectively.

### Measurement of ROS Levels

Intracellular levels of ROS were measured with dihydrorhodamine 123 (DHR123) purchased from Molecular Probes (USA). Briefly, cultured cells were collected by centrifugation and washed three times with PBS. Subsequently, the cells were adjusted to 2×10^7^ cells/ml. The cells were suspended with 50 mM sodium citrate buffer containing 2% glucose (pH 5.0). After incubation with 20 μM of DHR123 for 15 min at 30°C, the cells were exposed to H_2_O_2_ and incubated at 30°C with constant shaking (200 rpm). At specified intervals, the cell suspensions were harvested and examined by fluorescence microscopy using a Zeiss Axiovert 200 inverted microscope at excitation and emission wavelengths of 485 and 520 nm, respectively.

### Statistical Analysis

Statistical analyses were performed by two-tailed *t*-tests. Results are shown as mean ± standard deviation.

## Results and Discussion

### Trehalose Protects *S. boulardii* against Oxidant-Induced Cytotoxicity

**Fig. 1:**
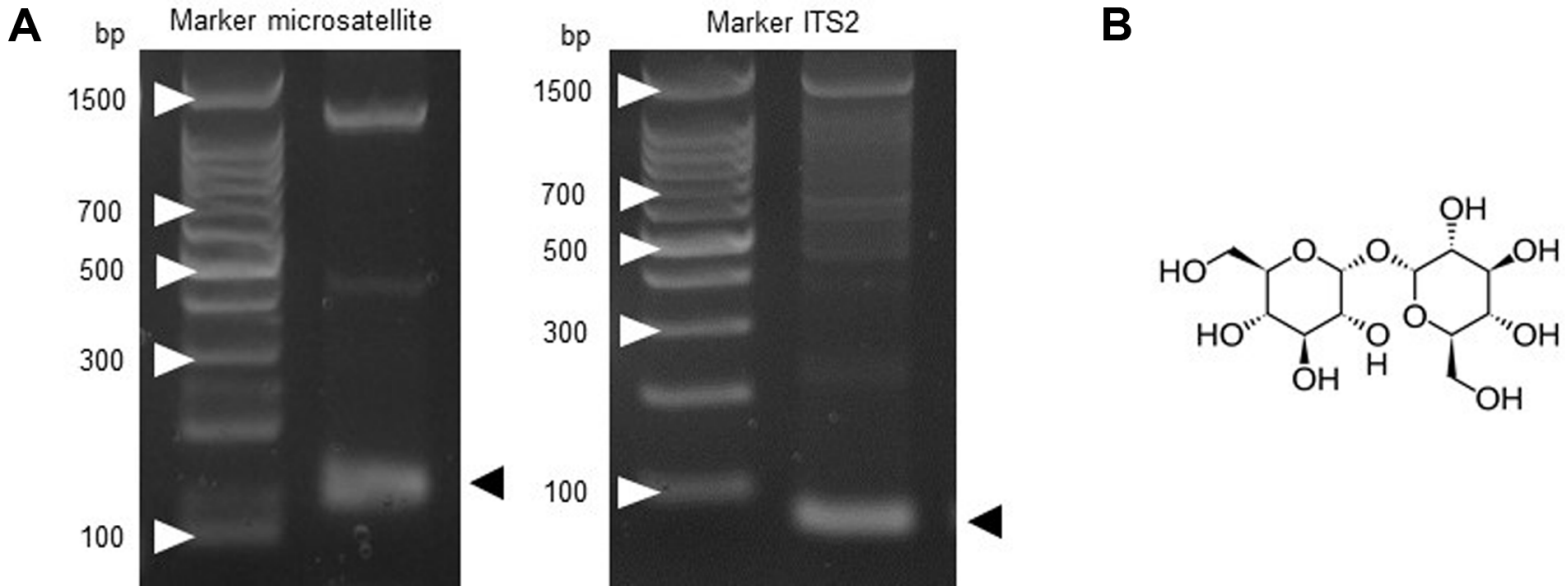
(**A**) Genetic identification of *Saccharomyces boulardii* by polymerase chain reaction (PCR)-based specific amplification of the 5.8S-ITS2 rDNA subunit region of genomic DNA. PCR amplification using *S. boulardii*-specific ITS2 primer pairs was performed as described in the Materials and Methods. Electrophoretic analysis of the PCR product of the ITS2 region in the nuclear rDNA of *S. boulardii* on a 2% agarose gel. (**B**) Chemical structure of trehalose.

**Fig. 2:**
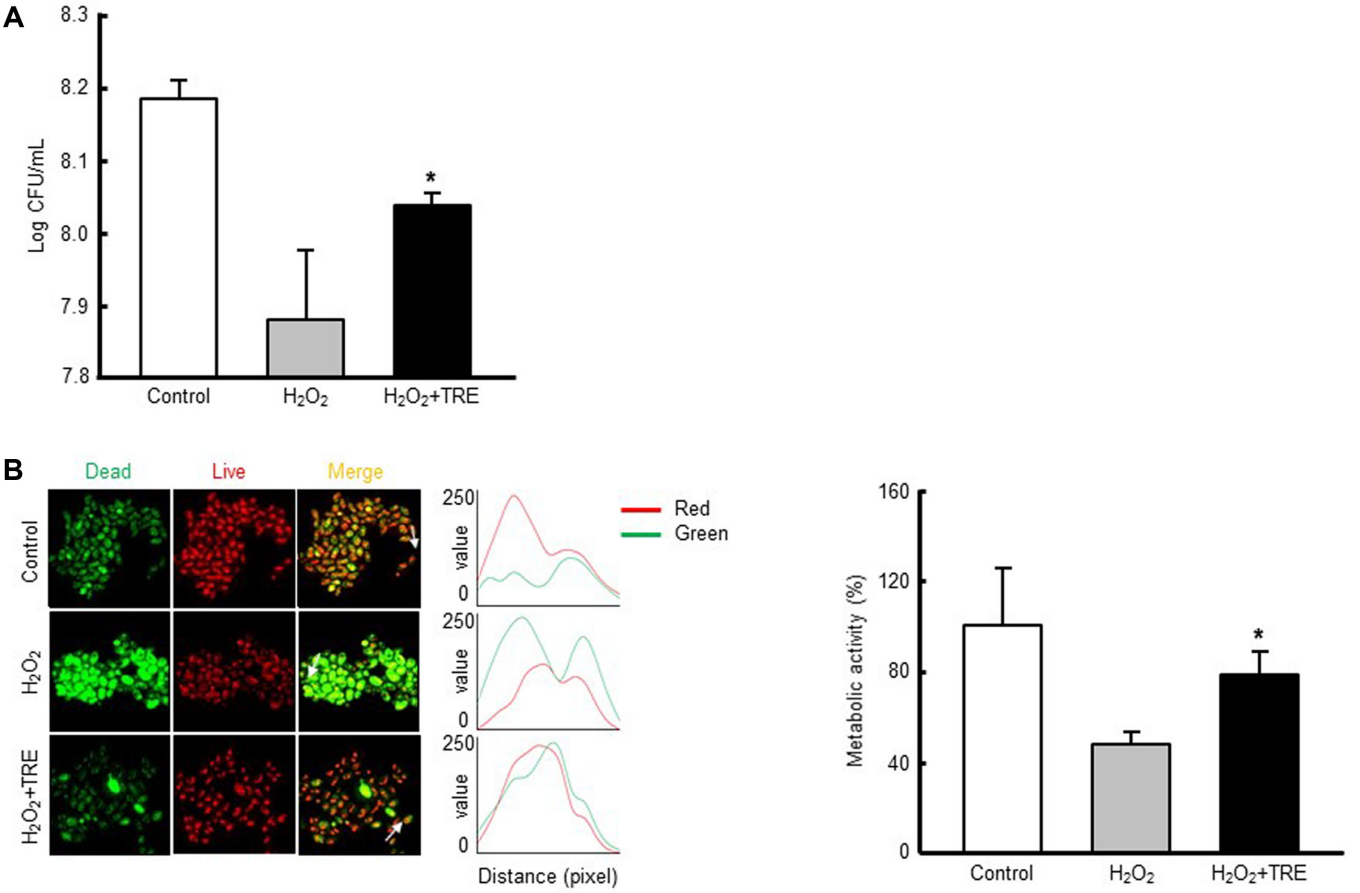
Protective effect of trehalose against oxidant-induced toxicity of *S. boulardii*. (**A**) Colony-forming unit assay. Exponentially growing yeast cells were preincubated with 1 M trehalose for 1 h, and subsequently incubated with 1 M H_2_O_2_ for a further 1 h. Following incubation, the yeast cells were spread onto yeast extract-peptone-dextrose agar plates and incubated for 24 h, then their viability was evaluated. (**B**) Metabolic activity of the yeast cells estimated by FUN-1 staining. Exponentially growing yeast cells were treated as described above, then incubated with FUN-1 for 30 min. After incubation, the yeast cells were washed three times with phosphatebuffered saline and observed by fluorescence microscopy. Representative images from at least three independent experiments are shown. Data are expressed as the ratio of red (λ = 575 nm) to green (λ = 535 nm) fluorescence. All values are presented as the mean ± standard deviation (SD) of three independent experiments. *p < 0.05 compared with H_2_O_2_-treated cells.

When the cells of a yeast such as *S. boulardii* (Fig. 1A) are exposed to hydrogen peroxide (H_2_O_2_), there is typically a pronounced decline in reproductive ability and an increase in cell mortality [29, 30]. However, antioxidant dietary supplements may protect probiotic microbes against H_2_O_2_- induced oxidative stress. Here, we first evaluated whether trehalose could protect *S. boulardii* against the oxidative stress induced by H_2_O_2_ (Fig. 1B). We performed a colonyforming unit assay to calculate the viability of *S. boulardii* cells that were pretreated with trehalose, then treated with H_2_O_2_ in liquid YPD medium. Exposure to 1 M H_2_O_2_ resulted in a significant decrease in cell viability as compared to non-H_2_O_2_-exposed control cells. This result was expected, since H_2_O_2_ generates the toxic and highly reactive hydroxyl radical, against which *S. boulardii* has no defense [21]. In contrast, pretreatment of the cells with 1 M trehalose before H_2_O_2_ treatment significantly increased their viability (Fig. 2A), suggesting that trehalose protected them against oxidant-induced cell death. To further evaluate the intracellular perturbations contributing to the growth inhibition of the cells observed after exposure to H_2_O_2_, we examined their metabolic activity using the FUN-1 stain. In cells stained with FUN-1, a higher red-to-green fluorescence ratio indicates a higher metabolic activity [27]. As shown in Fig. 2B, the cells exposed to H_2_O_2_ exhibited an impaired metabolic activity with diffuse green-yellow cytoplasmic fluorescence after FUN-1 staining. However, trehalose pretreatment resulted in a marked restoration of the H_2_O_2_-induced metabolic impairment, indicated by the presence of cylindrical red fluorescent structures in the cell vacuoles. Together, these results indicate that trehalose enhanced the survival of *S. boulardii* by protecting the microbe from the cell death associated with oxidative stress.

### Trehalose Inhibits the Oxidant-Induced PCD of *S. boulardii* in a YCA1-Dependent Manner

**Fig. 3:**
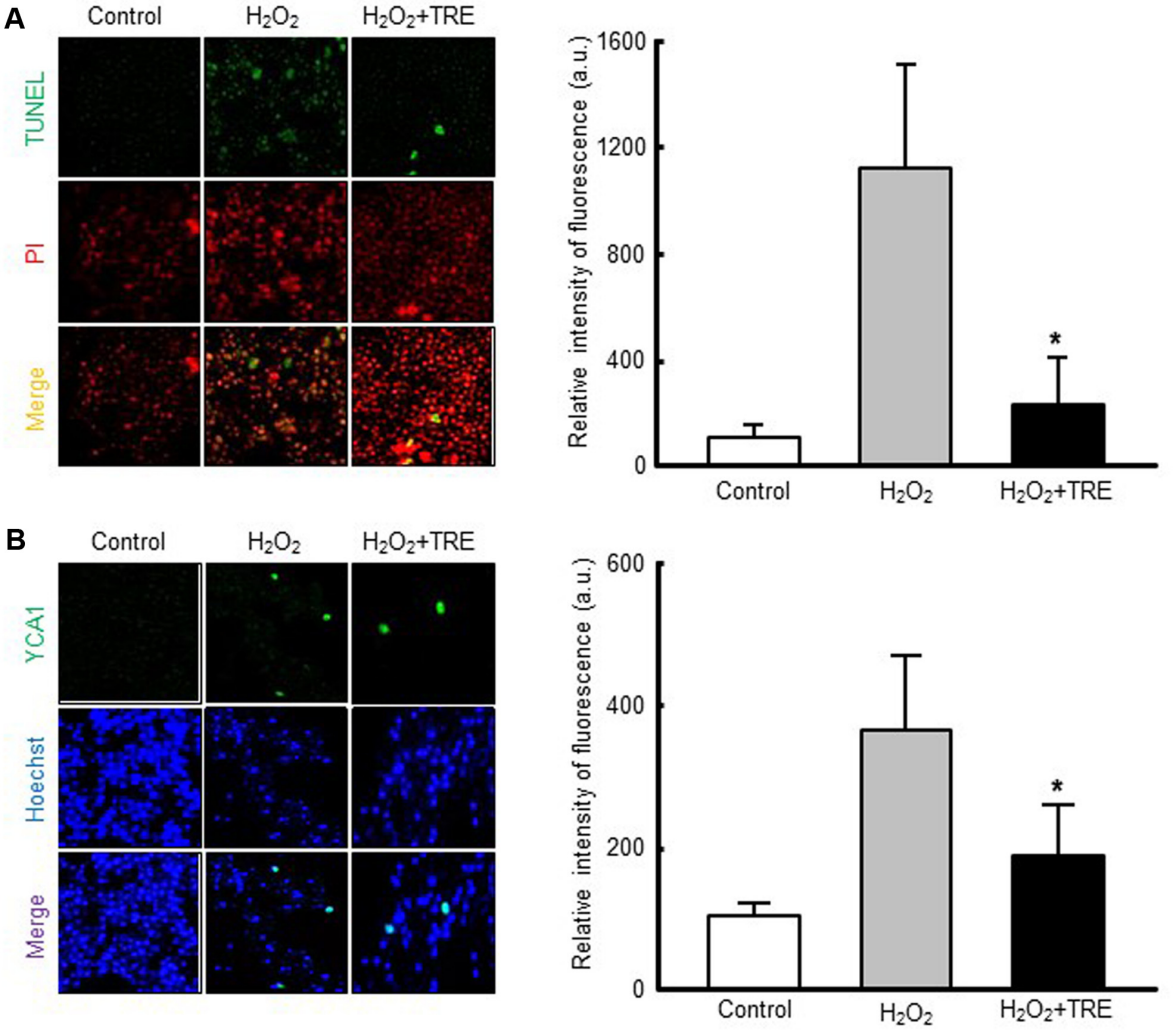
Protective effect of trehalose against the oxidant-induced programmed cell death of *S. boulardii* through inhibition of metacaspase YCA1. (**A**) Detection of apoptotic cells by deoxynucleotidyltransferase-mediated dUTP-biotin nick-end labeling (TUNEL) staining of the yeast cells treated with H_2_O_2_ with or without trehalose pretreatment. Staining for nuclear morphology was performed with propidium iodide. Representative images from at least three independent experiments are shown. Percentage of TUNEL-positive cells. At least 100 cells were examined per treatment. Data are the mean ± SD of three independent experiments. *p < 0.05 versus H_2_O_2_-treated cells. (**B**) Activated YCA1 caspase activity was detected using a CaspACE™ FITC-VAD-FMK *in situ* Marker, according to the manufacturer’s specifications. Nuclei were counterstained with Hoechst 33342. Representative images are shown from at least three independent experiments. Percentage of YCA1-positive cells. Data are presented as mean ± SD (*n* = 3 per a group). Asterisks represent a significant decrease in the proportion of YCA1-positive cells in the trehalose and H_2_O_2_-treated group compared to the H_2_O_2_-only group (*p* < 0.05).

Recent studies with yeasts including *S. boulardii* have demonstrated the occurrence of PCD associated with characteristic cell markers indicative of apoptosis in mammalian cells, including the condensation of chromatin, fragmentation of the nucleus, degradation of DNA, and the activation of caspase-like enzymatic activities [31]. In addition, yeasts have exhibited PCD in oxidant-induced environments, which is considered to be the main cause of reduced or lost cell viability in probiotic yeasts, such as *S. boulardii*. Therefore, to investigate the molecular mechanisms by which trehalose protected against cytotoxicity in *S. boulardii* exposed to oxidative stress, H_2_O_2_-exposed cells, with or without trehalose pretreatment were tested for PCD using a TUNEL assay. As shown in Fig. 3A, direct exposure of *S. boulardii* to H_2_O_2_ resulted in a TUNEL-positive phenotype, exhibiting yellow nuclear fluorescence as the result of superimposition of green and red fluorescence caused by simultaneous staining with TUNEL and propidium iodide. Furthermore, the percentage of cells displaying positive staining for TUNEL was higher in the cultures exposed to H_2_O_2_ than in the control cultures. However, the proportion of cells committed to PCD upon exposure to H_2_O_2_ declined sharply in the presence of trehalose, indicating that trehalose treatment arrested the PCD of *S. boulardii* in response to oxidative stress. Importantly, the decreased sensitivity to PCD after trehalose treatment was reflected in the cell viability results, suggesting that the inhibition of PCD by trehalose contributed to its protective effect against oxidant-induced cytotoxicity. We next examined the effect of trehalose on the modulation of PCD in *S. boulardii* after exposure to H_2_O_2_. In yeast, PCD can occur in a metacaspase-dependent or metacaspase-independent manner [32]. Thus, we asked whether the metacaspase YCA1 was responsible for the death of *S. boulardii* yeast cells. As shown in Fig. 3B, the activation of YCA1 was more pronounced when the cells were exposed to H_2_O_2_. However, when H_2_O_2_ was applied after trehalose pretreatment, the increased metacaspase activity seen in the cells was drastically reduced to levels comparable to those in control cells. This suggested that inactivation of the YCA1 metacaspase contributed, at least in part, to the protective effect of trehalose against the oxidant-induced PCD of *S. boulardii*. Taken together, our results proved that the extent of PCD in *S. boulardii* cells exposed to oxidative stress was significantly reduced by treating the organism with trehalose, which was reflected by increased cell survival under the oxidative environment.

### Trehalose Protects *S. boulardii* from Oxidative Stress by Reducing ROS Accumulation

**Fig. 4:**
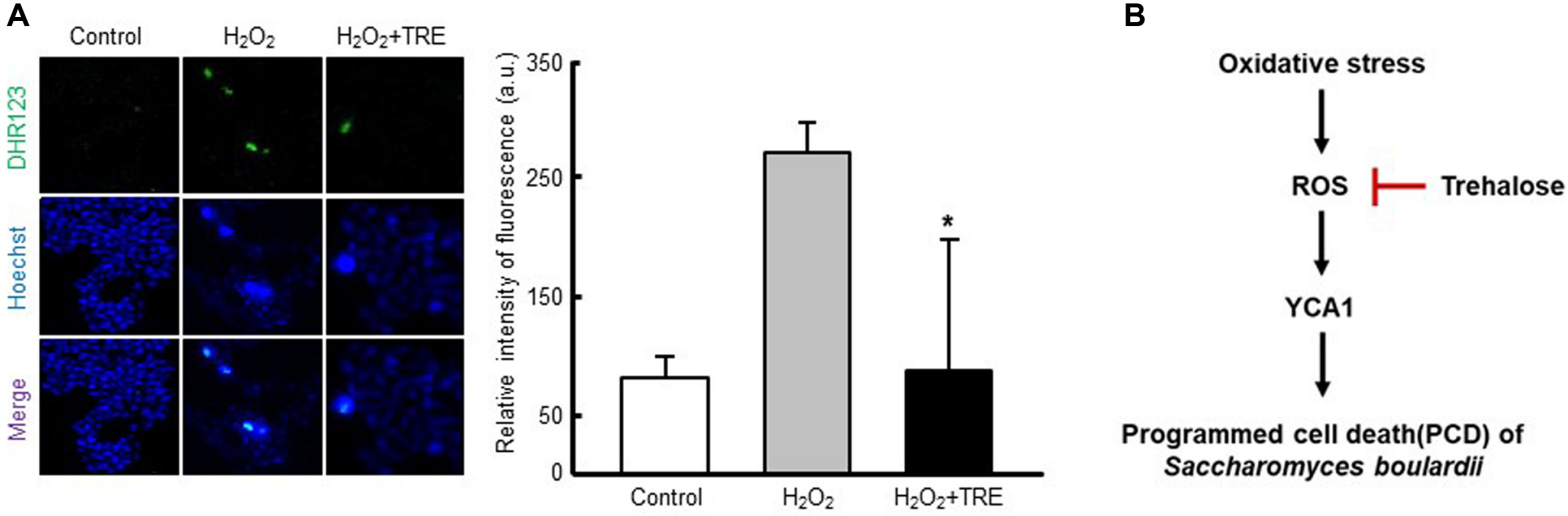
Protective effect of trehalose against intracellular ROS production in *S. boulardii* exposed to H_2_O_2_. (**A**) ROS detection by DHR123 staining. Exponentially growing yeast cells were incubated with 100 mM trehalose for 1 h, then incubated with 2 mM H_2_O_2_ for 1 h, followed by treatment with DHR123. Nuclei were counterstained with Hoechst 33342. Representative images from at least three independent experiments are shown. At least 100 cells were examined per treatment. Data represent the mean ± SD of the percentage of ROSpositive cells from three independent experiments. Asterisk represents a significant decrease in the proportion of ROS-positive cells in the trehalose and H_2_O_2_-treated groups compared to the H_2_O_2_-only group (*p* < 0.05). (**B**) Proposed mechanisms for the protective effect of trehalose against oxidative stress-induced cell death of *S. boulardii*. The non-reducing disaccharide trehalose enhances the cell viability of *S. boulardii* exposed to an oxidative environment by preventing ROS-mediated programmed cell death.

It is widely accepted that the generation of ROS in a wide range of organisms, including yeasts such as *S. boulardii*, is a key modulator of PCD [15]. Considering that H_2_O_2_ causes intracellular ROS production and exerts oxidative stress on the cells, it can be concluded that its toxicity is attributable to its ability to generate oxidative stress, which triggers PCD [33]. Since H_2_O_2_-induced oxidative stress adversely affects the growth of the probiotic microorganism, natural antioxidants should have a protective effect. Trehalose exhibits a wide range of biological effects [17, 24] and many of them have been attributed to its ROS-scavenging activity. Hence, to determine whether trehalose could increase the cellular tolerance to oxidative stress by ROS reduction, intracellular oxidation was measured using the DHR123 fluorescent probe. This probe is widely used to evaluate the enhancement of ROS after oxidative stress since, once inside the cell, it becomes susceptible to attack by ROS, which produces a more fluorescent compound [34]. As shown in Fig. 4A, after direct exposure of the yeast to H_2_O_2_, there was an increase in intracellular oxidation, in accord with the higher rate of PCD shown by the cells under this stress condition. However, when the cells were pretreated with trehalose, the levels of ROS produced in response to peroxide were almost 3-fold lower. This result suggests that trehalose has a high antioxidant capacity to eliminate the hydroxyl radicals formed by a Fenton reaction [21, 35], which is closely related to the amelioration of oxidative stress-mediated PCD in *S. boulardii*. Considering these results, the protection conferred by trehalose against intracellular oxidation appears to be directly correlated to the acquisition of oxidative stress tolerance, since the greatest increase in survival rate was reached during peroxide exposure.

In summary, the accumulation of ROS retarded the growth of *S. boulardii* when the organism was exposed to an oxidative environment. Oxidative stress followed by PCD was the major cause of the reduced cell viability of *S. boulardii*. Significantly, trehalose supplementation decreased intracellular ROS accumulation and YCA1-dependent PCD, while increasing the viability of *S. boulardii* cells exposed to H_2_O_2_ (Fig. 4B). These results suggest that the inclusion of trehalose, a commercially available and FDAapproved dietary supplement, together with *S. boulardii* may increase the viability of this probiotic yeast under the oxidative environment that occurs during cell growth. Therefore, the use of trehalose together with *S. boulardii* should improve its effectiveness, both as a probiotic and as a treatment for diarrhea and other gastrointestinal disorders.
